# Targeted metabolomic analysis of nitric oxide/L-arginine pathway metabolites in dementia: association with pathology, severity, and structural brain changes

**DOI:** 10.1038/s41598-019-50205-0

**Published:** 2019-09-24

**Authors:** Mariusz G. Fleszar, Jerzy Wiśniewski, Marzena Zboch, Dorota Diakowska, Andrzej Gamian, Małgorzata Krzystek-Korpacka

**Affiliations:** 1 Department of Medical Biochemistry, Wroclaw Medical University, 50-368 Wroclaw, Poland; 2 PORT Polski Ośrodek Rozwoju Technologii sp. z o.o., 54-066 Wrocław, Poland; 3 Research, Scientific, and Educational Center for Dementia Diseases of Wroclaw Medical University, 59-330 Ścinawa, Poland; 4 Department of Nervous System Diseases, Wroclaw Medical University, 51-618 Wroclaw, Poland

**Keywords:** Metabolomics, Molecular medicine, Alzheimer's disease

## Abstract

L-Arginine/NO pathway is altered in Alzheimer disease (AD). Its clinical relevance and pathway status in vascular dementia (VaD) are unknown. Using targeted metabolomics (a liquid chromatography-mass spectrometry) we assessed L-arginine, L-citrulline, dimethylamine (DMA), asymmetric dimethyl arginine (ADMA) and symmetric dimethylarginine (SDMA) in AD (n = 48), mixed-type dementia (MD; n = 34), VaD (n = 40) and non-demented individuals (n = 140) and determined their clinical relevance (the association with dementia pathology, cognitive impairment, and structural brain damage). L-Arginine, ADMA, L-arginine/ADMA, and L-citrulline levels were decreased in dementia and L-arginine, L-citrulline, age and sex were its independent predictors correctly classifying 91% of cases. L-Arginine and L-arginine/ADMA were differentiating between VaD and AD with moderate accuracy. L-Arginine, L-arginine/ADMA, SDMA, and DMA reflected structural brain changes. DMA and L-citrulline were elevated in patients with strategic infarcts and SDMA, L-arginine/ADMA, and DMA were independent predictors of Hachinski ischemic score. ADMA and SDMA accumulation reflected severity of cognitive impairment. In summary, L-Arginine/NO pathway is altered in neurodegenerative and vascular dementia in association with neurodegenerative and vascular markers of brain damage and severity of cognitive impairment.

## Introduction

Dementia becomes a growing clinical and socio-economical problem of aging societies. It is currently affecting approximately 50 million people worldwide but the number is predicted to increase to 152 million by 2050^1^. The correct and early diagnosis of dementia may improve the quality of life of affected patients as well as their families. However, therapeutic options for dementia are limited and unsatisfactory and no new drugs have been approved in recent 15 years^2^. Discerning the pathomechanisms at the molecular level is a prerequisite for identification of new potential biomarkers and therapeutic targets^2^.

Blood-based biomarkers are easier to measure than those derived from cerebrospinal fluid (CSF) and still believed to reflect the brain pathophysiology. Recently, metabolomics-based strategies are gaining attention as potential tools in the discovery of next-generation biomarkers^3^. Untargeted metabolomics studies have indicated that L-arginine/nitric oxide (NO) pathway is among those altered in Alzheimer disease (AD)^3^, a neurodegenerative disorder accounting for up to 80% of dementia cases^4^. The NO deficiency has been implicated in neurodegeneration by promoting endothelial dysfunction, accelerating formation and accumulation of amyloid peptides, reducing synaptic plasticity, activating microglia, and by evoking neuroinflammation^5^. The issue of pathway alteration in vascular dementia (VaD), the second most common type of dementia^4^, has not been addressed, even though the reduced NO availability is viewed as a possible common pathomechanism of VaD and AD^6^.

Brain homeostasis is secured by the activity of endothelial and neuronal isoforms of nitric oxide synthase (respectively eNOS and nNOS), catalyzing a two-step oxidation of L-arginine to NO and L-citrulline^7^. The NOS enzymes are negatively regulated by methylated derivatives of L-arginine. The methylation of L-arginine is catalyzed by class I and II protein arginine methyltransferases (PRMT), yielding, respectively, asymmetric and symmetric dimethylarginine (ADMA and SDMA). ADMA is a strong and SDMA a weak competitive inhibitor of NOS enzymes. Additionally, both dimethylarginines compete with L-arginine for its transporters, diminishing its intracellular availability. SDMA is mainly excreted by the kidneys while ADMA is catabolized to L-citrulline and dimethylamine (DMA) by dimethylarginine dimethylaminohydrolases (DDAH). L-citrulline can serve as a substrate for endogenous synthesis of arginine^7^.

The NO is a gaseous metabolite and its half-life is very short, making its accurate measurement a challenge. Instead, stable intermediates in NO metabolism such as L-arginine and ADMA have been evaluated. This study was designed to employ targeted metabolomics and our newly developed assay^8^ to evaluate the association of a panel of NO-related metabolites, namely, L-arginine, L-citrulline, ADMA, SDMA, and DMA with dementia, its probable pathology (vascular or neurodegenerative), severity of loss of cognitive function, structural changes in the brain as shown by the magnetic resonance imaging (MRI), and with brain ischemia. We demonstrated, for the first time, the alterations in NO-related metabolites in VaD, their distinct association patterns with the severity of dementia, brain atrophy and ischemia as well as their dependence on dementia pathology.

## Results

### Intermediates of NO metabolism and dementia

**Figure 1:**
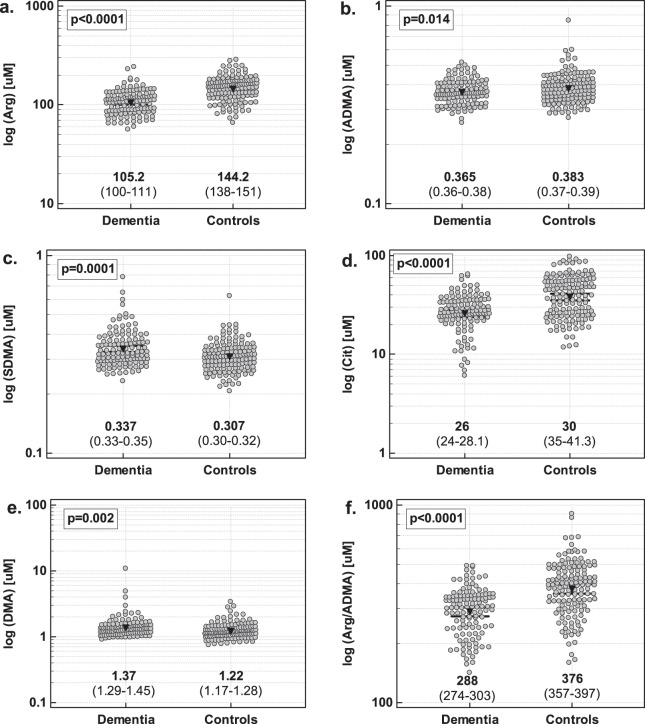
Intermediates of NO metabolism in patients with and without dementia: (**a**) arginine; (**b**) ADMA; (**c**) SDMA; (**d**) citrulline; (**e**) DMA; (**f**) Arg/ADMA. Data analyzed using t-test for independent samples. Geometric mean values and 95% confidence interval are given below the dot-plots and presented graphically by inverse triangles and whiskers.

As compared to control individuals, patients with dementia had significantly higher SDMA and DMA concentrations and lower ADMA, arginine-to-ADMA ratio (Arg/ADMA), L-arginine, and L-citrulline (further referred to as arginine and citrulline) (Fig. 1).

Since there was significant difference in age distribution between groups, data were re-analyzed using analysis of covariance (ANCOVA) with age as a covariate. With age differences accounted for, arginine (p = 0.014 for age and p < 0.001 for dementia), ADMA (p = 0.045 for age and p = 0.002 for dementia), Arg/ADMA (p = 0.001 for age and p = 0.004 for dementia), and citrulline (p = 0.454 for age and p < 0.001 for dementia) remained significantly different between controls and patients but the differences in SDMA (p < 0.001 for age and p = 0.817 for dementia) and DMA (p = 0.060 for age and p = 0.349 for dementia) lost their significance.

**Figure 2:**
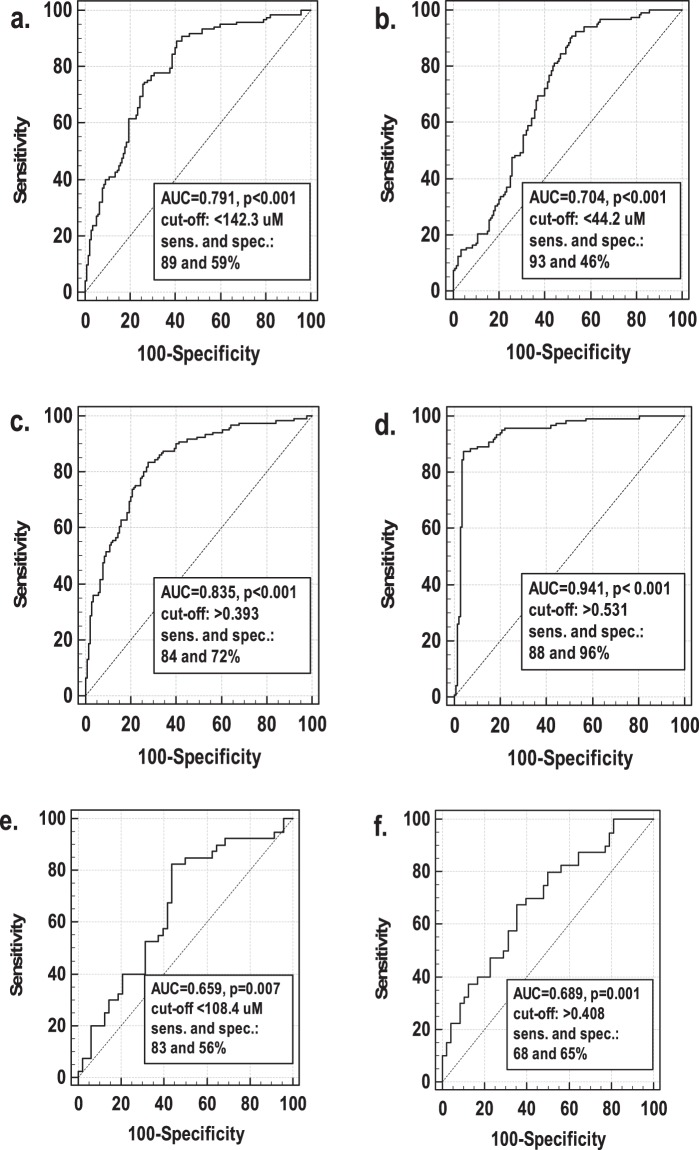
Receiver operating characteristics (ROC) curves: (**a**) arginine as dementia predictor; (**b**) citrulline as dementia predictor; (**c**) arginine and citrulline combined as dementia predictor; (**d**) model including log(arginine), log(citrulline), age and sex as dementia predictor; (**e**) arginine as a differential marker for vascular and Alzheimer dementia; (**f**) model including log(arginine) and BMI as a differential marker for vascular and Alzheimer dementia. AUC, area under ROC curve; sens., sensitivity; spec. specificity.

In logistic regression with dementia as a dependent variable and log (arginine), log (ADMA), log (citrulline), age, and sex as independent variables, the predictive model was build, which correctly classified 91% of cases. The model included log (arginine) (regression coefficient b = −5.72, p = 0.004), log (citrulline) (b = −3.83, p = 0.006), age (b = 0.20, p < 0.001), and sex (b = 0.96, p = 0.025) as significant predictors of dementia. The receiver operating characteristics (ROC) curve analysis was employed to calculate the overall accuracy (area under ROC curve expressed as %) as well as sensitivities and specificities of the model in comparison with individual and combined determination of arginine and citrulline. The overall accuracy of individual assessment of citrulline and arginine was 70% and 79%, respectively, and both markers were characterized by satisfactory sensitivity but poor specificity (Fig. 2a,b). Their combined determination improved overall accuracy to 84% and specificity to 72% (Fig. 2c). The model including age and sex in addition to arginine and citrulline had the overall accuracy of 94% with 88% sensitivity and 96% specificity (Fig. 2d). If arginine and ADMA were replaced by Arg/ADMA (which could not be co-evaluated with arginine and ADMA, based on which it is calculated, due to multicollinearity problems), it was retained in the model instead of arginine and the model had very similar characteristics.

### The association of intermediates of NO metabolism with type of dementia

**Figure 3:**
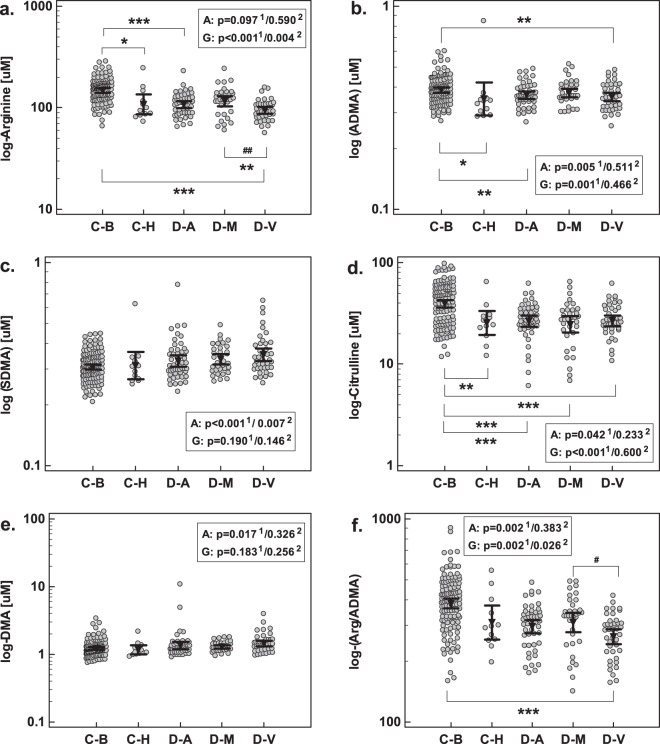
Intermediates of NO metabolism in study population with respect to dementia pathology: (**a**) arginine; (**b**) ADMA; (**c**) SDMA; (**d**) citrulline; (**e**) DMA; (**f**) Arg/ADMA. Data analyzed using analysis of covariance (ANCOVA) with age as a covariate either in a whole population with significance of age (A) and group (G) effect marked with “1” in superscript. The same analysis was repeated exclusively on dementia patients and the significance of age and group effects was marked as “2” in superscript. Geometric mean values are presented graphically by inverse triangles and 95% confidence interval around mean by whiskers. C-B, controls: blood donors; C-H, controls: non-demented patients; D-A, Alzheimer disease; D-M, mixed-type dementia; D-V, vascular dementia. *p < 0.05; **p < 0.01; ***p < 0.001 (for whole cohort analysis); ^##^p < 0.01 (for dementia cohort analysis).

The NO pathway-associated metabolites were assessed in two types of controls: healthy blood donors and age-matched individuals diagnosed for dementia or brain tumors due to unexplained headaches, memory problems or dizziness, in whom, however, neither dementia, mild cognitive loss nor serious somatic disease were confirmed (referred to as non-demented patients). After accounting for disparity in age, levels of arginine, ADMA, and citrulline were significantly lower in non-demented patients as compared to healthy individuals (Fig. 3). The mean metabolite concentrations as well as results of an unadjusted analysis are presented in Table 1.

**Table 1:** Comparison of control and dementia groups.

Parameter	Controls		Dementia			P value
	Blood donors	Non-dementia patients	Alzheimer disease	Mixed-type dementia	Vascular dementia	
N	128	12	48	34	40	
Sex (F/M)	90/38	10/2	34/15	22/11	21/19	0.171^χ2^
Age [yrs.]^e^	57.9 ± 6.2	70.2 ± 12.3^a^	74.9 ± 8^a^	75.5 ± 7.4^a^	72.8 ± 8.4^a^	<0.0001^A^
Education [yrs.]^e^	—	11 ± 3.2	9.5 ± 3.1	8.4 ± 3.6	9.5 ± 2.7	0.077^A^
MNA^f^	—	14 (13.75–14)	13 (13–13)^b^	13 (13–13)^b^	13 (13–13)^b^	<0.001^K^
BMI [kg/m^2^]^e^	—	25.2 ± 4.2	27.1 ± 4.1	27.5 ± 4.1	29 ± 5.2^b^	0.047^A^
MMSE^f^	—	29 (28.75–29.25)	17 (14–20.5)^b^	19 (17–22)^b,c^	19 (15–21)^b^	<0.0001^K^
CDR^f^	—	0 (0–0)	1 (1–2)^b^	1 (1–2)^b^	2 (1–2)^b^	<0.0001^K^
WMH^f^	—	1 (1–1)	1 (1–2)	2 (2–3)^b,c^	3 (2–3)^b–d^	<0.0001^K^
GCA^f^	—	1 (1–1)	2 (2–2)^b^	2 (2–2)^b^	2 (2–3)^b,c^	<0.0001^K^
MTA^f^	—	1 (0–1)	2 (2–2.75)^b^	2.25 (2–3)^b^	2.5 (2–3)^b^	<0.0001^K^
HIS^f^	—	2 (1–2.25)	3 (2–3)^b^	5 (5–6)^b,c^	7 (7–8)^b–d^	<0.0001^K^
Arginine [µM]^g^	148.2 (141–155)	108 (86.5–135)^a^	107.5 (99.7–116)^a^	116 (104–130)^a^	94.3 (87.7–101)^a,c,d^	<0.001^A^
ADMA [µM]^g^	0.39 (0.38–0.4)	0.35 (0.29–0.42)	0.37 (0.35–0.38)	0.38 (0.36–0.39)	0.36 (0.34–0.38)^a^	0.018^A^
SDMA [µM]^g^	0.31 (0.30–0.32)	0.31 (0.27–0.36)	0.33 (0.31–0.35)	0.33 (0.32–0.35)	0.35 (0.31–0.36)^a^	0.001^A^
Citrulline [µM]^g^	39.5 (36.3–42.9)	25.5 (19.3–33.5)^a^	26.4 (23.3–30)^a^	24.5 (20.3–29.6)^a^	26.8 (23.7–30.2)^a^	<0.001^A^
DMA [µM]^g^	1.23 (1.17–1.28)	1.17 (1–1.36)	1.35 (1.2–1.52)	1.3 (1.22–1.37)	1.45 (1.32–1.6)^a^	0.013^A^
Arg/ADMA^g^	383.4 (363–405)	309 (255–375)^a^	294.8 (274–317)^a^	309.7 (277–346)^a^	263.3 (243–286)^a^	<0.001^A^

N, number of participants; F/M, female-to-male ratio; yrs., years; ADMA, asymmetric dimethylarginine; SDMA, symmetric dimethylarginine; DMA, dimethylamine; Arg/ADMA, arginine-to-ADMA ratio; MNA, the Mini Nutritional Assessment score; BMI, body mass index; MMSEs, the Mini Mental State Examination score; CDR; the Clinical Dementia Rating; WMH, the Fazekas scale for white matters hyperintensities; GCA, the Global Cortical Atrophy scale; MTA, the Medial Temporal Lobe Atrophy; HIS, the Hachinski Ischemic Scale; ^χ2^Chi-squared test; ^A^one-way analysis of variance; ^K^Kruskal-Wallis H test; ^a^significantly different from blood donors; ^b^significantly different from non-dementia control group; ^c^significantly different from Alzheimer disease, ^d^significantly different from mixed-type dementia; ^e^data presented as mean ± standard deviation; ^f^data presented as medians with interquartile range; ^g^data presented as geometric mean with 95% confidence interval.

None of evaluated parameters differed significantly between demented patients (any type of dementia) and non-demented patients (Fig. 3, Table 1).

Following adjustment to disparity in age, patients with Alzheimer dementia had decreased arginine, ADMA, and citrulline as compared to healthy individuals and patients with mixed-type dementia had decreased citrulline. Patients with vascular dementia had decreased arginine, ADMA, citrulline, and Arg/ADMA as compared to healthy controls and decreased arginine and Arg/ADMA as compared to patients with mixed-type dementia (Fig. 3, Table 1).

To confirm more pronounced arginine depletion in vascular dementia, a logistic regression was conducted with VaD/AD as dependent variable (VaD encoded by 1 and AD as 0) and log (arginine), age, sex, BMI, and MMSE as independent variables. Log (arginine) (b = −6.35, p = 0.008) and BMI (b = 0.12, p = 0.028) were found to be independent predictors of VaD. The model correctly classified 61.4% of cases. The overall accuracy of combined arginine and BMI assessment was 69% and sensitivity and specificity were 68 and 65%, respectively (Fig. 2f). Arginine alone had an accuracy of 66% and sensitivity and specificity of 83 and 56%, respectively (Fig. 2e). The model, in which arginine was replaced with Arg/ADMA, yielded very similar results (66% of correctly classified cases and 66% overall accuracy).

### The association of intermediates of NO metabolism with cognitive loss

#### Mini-Mental State Examination (MMSE)

**Figure 4:**
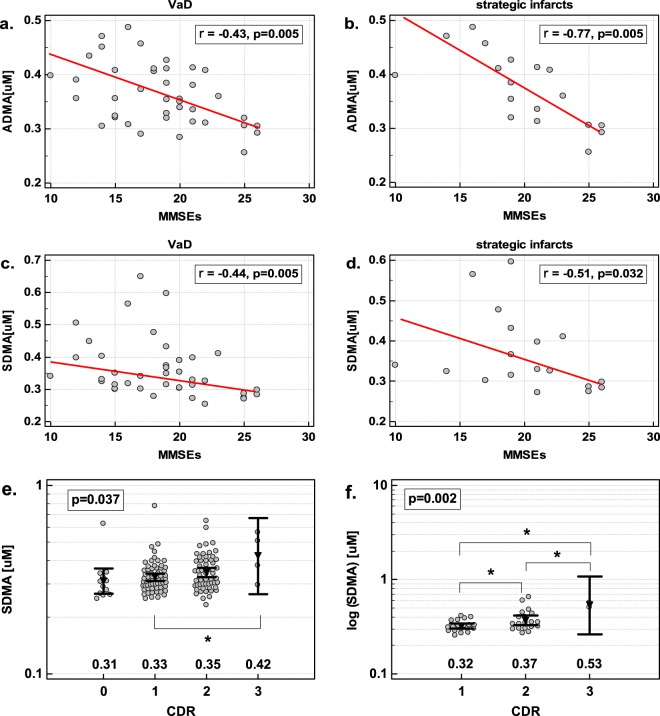
The association of dimethylarginines concentration in serum with the degree of cognitive loss: (**a**) ADMA and MMSEs in patients with vascular dementia; (**b**) ADMA and MMSEs in patients with strategic infarcts; (**c**) SDMA and MMSEs in patients with vascular dementia; (**d**) SDMA and MMSEs in patients with strategic infarcts; (**e**) SDMA and CDR in all patients (including non-demented controls); (**f**) SDMA and CDR in patients with vascular dementia. Data presented as Spearman correlation (panels a–d) or one-way ANOVA with geometric mean reported below the dot-plots; significant between-group differences are marked with connectors with asterisk.

In VaD, ADMA and SDMA were inversely correlated with the MMSE score, indicating that VaD patients with a high degree of intellectual impairment displayed higher ADMA and SDMA levels than patients with a low degree of cognitive impairment (Fig. 4a,b). The Arg/ADMA displayed a similar tendency (ρ = −0.31, p = 0.056). The association was stronger in a subset of patients with strategic infarcts (Fig. 4c,d). None of the other metabolites showed significant association with the MMSE.

In the age-, sex-, and BMI-adjusted analysis, log (ADMA), but not log (SDMA), was an independent predictor of the MMSE score in VaD, explaining 22% in its variability (b = −28, p = 0.002, r_p_ = −0.47). In the analysis adjusted to those factors but restricted to VaD patients with strategic infarcts, log (ADMA) as an independent predictor explained 52% in MMSE variability (b = −38.9, p < 0.001, r_p_ = −0.72).

#### Clinical Dementia Rating (CDR)

The SDMA was positively associated with the CDR score in all patients (ρ = 0.23, p = 0.007) but markedly more in VaD (ρ = 0.41, p = 0.009) and patients with strategic infarcts (ρ = 0.50, p = 0.034) (Fig. 4e,f).

In the age-, sex-, and BMI-adjusted analysis including dementia type (with AD and VaD as dummy variables), log (SDMA) was an independent predictor of the CDR score in all patients, explaining 5% of its variability (b = 1.35, p = 0.016, r_p_ = 0.22). In the age-, sex-, BMI-adjusted analysis in VaD patients, log (SDMA) as an independent predictor explained 25% of the CDR variability (b = 3.05, p = 0.001, r_p_ = 0.50). In the analysis restricted to patients with strategic infarcts, log (SDMA) explained 42% variability in the CDR (b = 3.81, p = 0.003, r_p_ = 0.65).

### The association of intermediates of NO metabolism with the structural changes in the brain

#### Global cortical atrophy (GCA)

The DMA (ρ = 0.23, p = 0.018) and SDMA (ρ = 0.24, p = 0.014) positively, although weakly, correlated with the GCA score in a whole cohort. In the age-, sex-, and BMI-adjusted analysis, DMA and age, but not SDMA, were independent predictors of the GCA score, explaining 13% in its variability (b = 0.80, p = 0.048, r_p_ = 0.19 for DMA and b = 0.021, p = 0.002, r_p_ = 0.29 for age).

**Figure 5:**
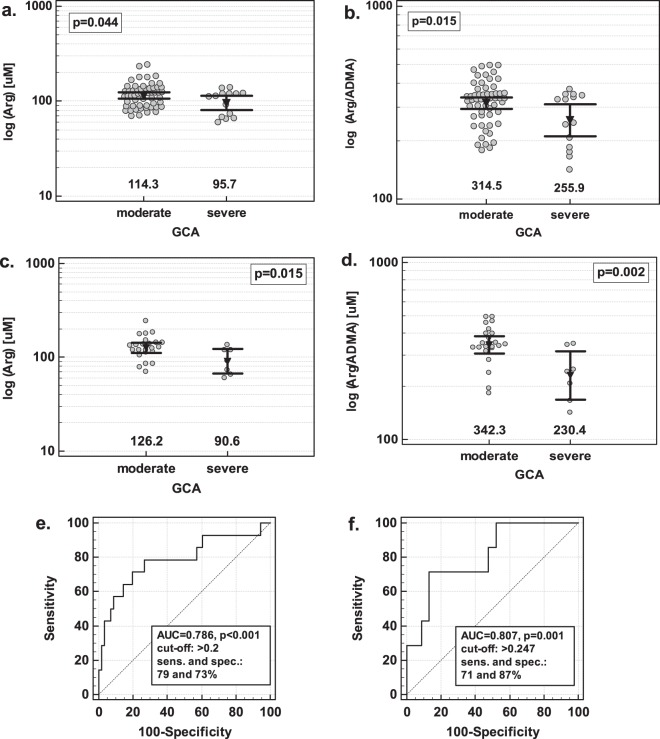
The association between serum concentration of intermediates of NO metabolism and the degree of global cortical atrophy (GCA): (**a**) arginine in AD + MD patients; (**b**) Arg/ADMA in AD + MD patients; (**c**) arginine in MD patients; (**d**) Arg/ADMA in MD patients; (**e**) receiver operating characteristic (ROC) curve for Arg/ADMA and age as predictors of severe GCA in AD + MD patients (**f**) ROC curve for Arg/ADMA as a severe GCA predictor in MD patients. AD, Alzheimer disease; MD, mixed-type dementia; AUC, area under ROC curve; sens., sensitivity; spec. specificity. Data analyzed using t-test for independent samples (panels a–d) with numbers below the dot plots indicating the geometric mean values presented also graphically by inverse triangles with whiskers (95% confidence interval around mean).

Arginine and Arg/ADMA were associated with severe brain atrophy as they were significantly decreased in patients with GCA score 3. The association could be observed in a subgroup of AD + MD patients (neurodegenerative pathology) (Fig. 5a,b) and in MD patients (Fig. 5c,d). In VaD patients, however, there was an opposite tendency and Arg/ADMA was insignificantly higher in patients with severe atrophy (p = 0.091).

In the age-, sex-, and BMI-adjusted analysis, log (Arg/ADMA) (b = −5.3, p = 0.046) and age (b = 0.14, p = 0.022) were independent predictors of severe global cortical atrophy in a group of AD + MD patients, correctly classifying 83% of cases. Their overall accuracy as severe brain atrophy indicator was 79% and sensitivity and specificity was 79% and 73%, respectively (Fig. 5e). If arginine was entered into the model instead of Arg/ADMA, only age was retained in the predictive model.

In the age-, sex-, and BMI-adjusted analysis, log (Arg/ADMA) (b = −10, p = 0.013) was an independent predictor of severe global cortical atrophy in MD patients, correctly classifying 80% of cases. Its overall accuracy as severe atrophy indicator was 81% and sensitivity and specificity was 71% and 87% (Fig. 5f). If arginine was entered into the model instead of Arg/ADMA, arginine was an independent predictor, allowing for correctly classifying 83% of cases but its overall accuracy was 78%.

#### Medial temporal lobe atrophy (MTA)

Exclusively in a subgroup of patients with strategic infarcts, the concentrations of DMA (ρ = 0.46, p = 0.052) tended to positively correlate with the average MTA score (see Supplementary Fig. S1). In the age-, sex-, and BMI-adjusted analysis, log (DMA) (b = 1.31, p = 0.0.27, r_p_ = 0.52) was an independent predictor of the MTA score, explaining 27% of its variability in patients with strategic infarcts. None of the other metabolites differed significantly with respect to the MTA.

#### Fazekas scale for white matters hyperintensities (WMH)

None of metabolites, either in a whole cohort or in subgroups based on dementia type, differed significantly with respect to the Fazekas score for white matter hypersensitivities.

### The association of intermediates of NO metabolism with the vascular changes in the brain

#### Strategic infarcts

**Figure 6:**
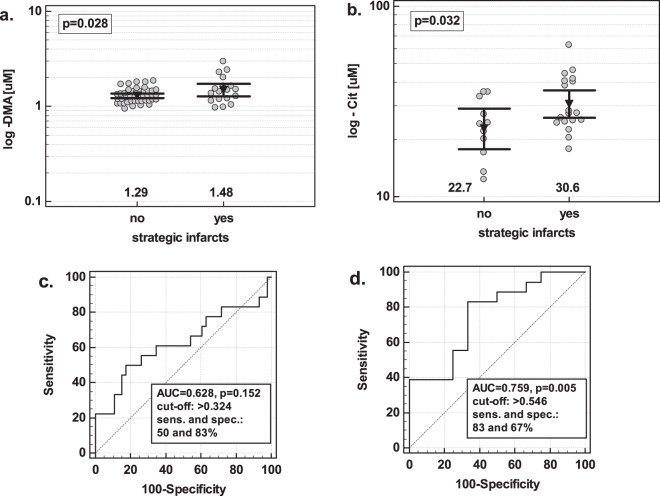
The association between serum concentration of intermediates of NO metabolism and the presence of strategic infarcts. (**a**) DMA in VaD + MD patients; (**b**) citrulline in VaD patients. Data analyzed using t-test for independent samples. Numbers below the dot plots indicate the mean values. Mean values are additionally presented graphically by inverse triangles and 95% confidence interval around mean by whiskers.

The concentrations of DMA were significantly higher in patients with strategic infarcts than without if analyzed in a group of VaD + MD patients. In the age-, sex-, and BMI-adjusted analysis, log (DMA) (b = 6.24, p = 0.039) was an independent predictor of strategic infarcts, correctly classifying 78% of cases. Its overall accuracy was 63% and sensitivity and specificity were 50 and 83%, respectively (Fig. 6a,c).

Citrulline concentrations were significantly higher in VaD patients with infarcts. In the age-, sex-, and BMI-adjusted analysis, log (citrulline) (b = 6.54, p = 0.041) was an independent predictor of strategic infarcts, correctly classifying 70% of cases. Its overall accuracy was 76% and sensitivity and specificity were 83 and 67%, respectively (Fig. 6b,d).

#### Hachinski ischemic score (HIS)

**Figure 7:**
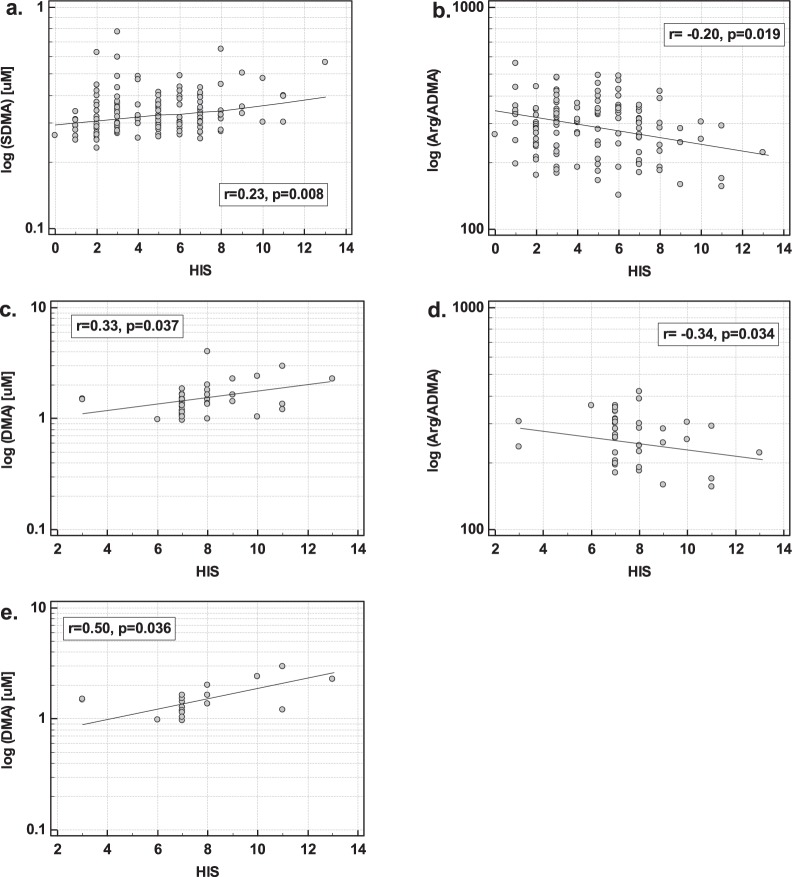
The association between intermediates of NO metabolism and Hachinski ischemic score (HIS). (**a**) SDMA and HIS in all patients; (**b**) Arg/ADMA and HIS in all patients; (**c**) DMA in VaD patients; (**d**) Arg/ADMA and HID in VaD patients; (**e**) DMA in patients with strategic infarcts. Data presented as Pearson correlation coefficients. VaD, vascular dementia.

The SDMA positively and Arg/ADMA negatively correlated with the HIS in the whole cohort of patients (Fig. 7a,b). In the age-, sex-, and BMI-adjusted analysis, both log (SDMA) (b = 5.53, p = 0.017, r_p_ = 0.21) and log (Arg/ADMA) (b = −3.74, p = 0.033, r_p_ = −0.19) and additionally sex (b = 1, p = 0.013, r_p_ = 0.22) and BMI (b = 0.15, p < 0.001, r_p_ = 0.29) were independent predictors of the HIS score, explaining 19% in its variability.

In VaD, DMA positively and Arg/ADMA negatively correlated with the HIS (Fig. 7c,d). In the age-, sex-, and BMI-adjusted analysis, both log (DMA) (b = 4.43, p = 0.029, r_p_ = 0.36) and log (Arg/ADMA) (b = −5.04, p = 0.039, r_p_ = −0.34) and additionally age (b = −0.07, p = 0.028, r_p_ = −0.36) were independent predictors of the HIS score, explaining 29% in its variability.

In patients with strategic infarcts, DMA was the only NO-associated metabolite correlating with HIS (Fig. 7e). In the age-, sex-, and BMI-adjusted analysis, log (DMA) (b = 9.09, p = 0.036, r_p_ = 0.50) was an independent predictor of the HIS, explaining 25% in its variability.

### The association of intermediates of NO metabolism with the nutritional status of patients

Nutritional status was assessed using body mass index (BMI) and the Mini Nutritional Assessment (MNA).

Exclusively in patients with MD, the BMI inversely correlated with citrulline (r = −0.49, p = 0.003), ADMA (r = −0.35, p = 0.041), and SDMA (r = −0.36, p = 0.037). In the age-, sex, and MMSE-adjusted analysis, BMI remained significantly associated with all.

None of the parameters correlated with the MNA score.

### Interplay between intermediates of NO metabolism

Concentrations of intermediates associated with NO metabolism were interrelated but there were differences between healthy individuals and dementia patients as well as among dementia patients with respect to the dementia type (for details see Supplementary Fig. S2). In multivariate analysis, DMA was an independent predictor of arginine but exclusively in healthy individuals. The SDMA alone was an independent predictor of ADMA in all patients and in combination with citrulline and arginine in AD. The DMA and ADMA were independent predictors of SDMA in all patients. Citrulline concentrations were predicted by ADMA in healthy controls and in AD. The DMA was predicted by SDMA in all patients but the association was stronger in VaD (for details see Supplementary Table S1).

## Discussion

Here, we used targeted metabolomics to evaluate the clinical and diagnostic relevance of intermediates of arginine/NO metabolism, the pathway identified as altered in previous studies employing untargeted approach^3^. Unlike earlier research, this study explores metabolites beyond arginine and ADMA and analyzes dementia beyond AD. Corroborating previous findings^9–12^, arginine in our AD patients was significantly decreased. However, lack of significant changes^13–16^ and occasionally an elevation^17,18^ have been observed as well. Those discrepancies might result from small number of analyzed cases in some of those studies and/or lack of brain imaging supporting the diagnosis of dementia pathology. We demonstrated that arginine was more noticeably diminished in association with vascular pathology. To the best of our knowledge, arginine in VaD has not been evaluated but, in patients with acute ischemic stroke, arginine decrease has been associated with early neurologic deterioration as well as poor outcome. Moreover, its concentrations in CSF and plasma have been the lower the higher the volume of infarct^19^. Furthermore, arginine intervention within an hour from the onset of stroke-like episodes in patients with MELAS syndrome have caused normalization of clinical symptoms and withdrawal of pathological changes visualized by MRI^20^. More recently, a positive clinical response to arginine has been reported also in a larger pediatric cohort of metabolic stroke patients due to mitochondrial diseases beyond the MELAS^21^. In patients of neurodegenerative pathology arginine was inversely associated with neurodegenerative measures – its concentrations were significantly lower in AD and MD patients with severe brain atrophy (expressed in terms of the GCA score). This finding supports the earlier notion on arginine decrease being associated with dementia progression, which was based on the observation that arginine concentrations are more noticeably decreased in AD than in mild cognitive impairment (MCI)^10,11^. Interestingly, the authors reporting an increase in arginine have observed its further rise with progression from the MCI to AD^17,18^. Similarly to others in AD^13^, we did not observe arginine correlation with the degree of intellectual deficiency regardless the scale used for its evaluation (MMSEs or CDR). Also population-based studies on elders have not shown arginine concentrations to be associated with subjective or objective memory impairment^22^.

Our observation of decreased concentrations of ADMA in AD is unexpected and contradicts the previous findings showing either ADMA elevation^23,24^ or lack of significant alterations^11,13,16^. Noteworthy, contradicting results concerning ADMA have even been reported by the same group^11,24^ stressing ADMA level dependence on cohort characteristics. The ADMA accumulation is expected in the conditions of oxidative stress and inflammation as a result of the up-regulated expression of PRMT enzymes (increased rate of ADMA synthesis) and the down-regulated expression of DDAH enzymes (decreased rate of ADMA degradation)^7^. However, Morales *et al*.^25^ challenged the notion demonstrating that PRMT1, a major class I isoform of the enzyme responsible for ADMA synthesis, is inhibited under oxidative stress condition. Similarly, Lim *et al*.^26^ showed that both the expression of class I PRMTs and the synthesis of ADMA-containing polypeptides were diminished in oxidatively premature senescent fibroblasts. The PRMT5, a major class II enzyme, was less strongly down-regulated and the net synthesis of SDMA-containing polypeptides was increased, implying that other class II PRMTs might be up-regulated by oxidative challenge. Altogether, these findings might explain both significantly lower ADMA in our patients and the disparity between ADMA and SDMA. The ADMA decrease has been observed also in other neurodegenerative conditions^27^. Moreover, a neuroprotective role for ADMA in Parkinson’s disease has even been suggested^28^.

In the present study, ADMA was decreased also in VaD. While no previous reports on this metabolite in vascular dementia could be found, ADMA association with stroke is controversial with both increased and unaltered concentrations being reported (reviewed in^29^). If observed, ADMA elevation has not predicted all-cause mortality^30^ or adverse clinical events^31^ after acute stroke^30^ or correlated with stroke severity^32^, implying a less important role for ADMA in stroke^30^ than in the primary cardiovascular disease^33^. Here, although lower than in controls, ADMA accumulation in dementia was associated with unfavorable characteristics. It was an independent predictor of the MMSEs score, reflecting an increasing cognitive deterioration in VaD patients. The association was particularly strong in patients who suffered from strokes in brain regions strategic for cognitive impairment. In agreement, increasing ADMA concentrations have been linked with subjective memory loss^22^ and with the risk of subclinical vascular brain injury^34^ in older individuals without dementia in a population-based study. Correspondingly, others have declared ADMA an AD marker relevant for the disease progression based on its accelerated accumulation in AD as compared to MCI^35^.

The arginine-to-ADMA ratio (Arg/ADMA) is believed to better reflect NO bioavailability than arginine or ADMA alone. Congruently, Arg/ADMA, but not arginine or ADMA individually, have correlated with the severity of ischemic stroke^32^ or with the intima-media thickness, a risk factor for atherosclerosis^36^. In our study, Arg/ADMA was an independent predictor of dementia, but unlike arginine, which was decreased in both, Arg/ADMA was significantly reduced in dementia of vascular pathology, of which it was an independent predictor. In line with beneficial role attributed to NO^5^, its diminished availability in VaD patients tended to reflect the degree of cognitive function impairment. Although not decreased in general, a noticeably reduced bioavailability of NO accompanied severe brain atrophy in demented patients with neurodegenerative component. It was also associated with an increasing ischemia, particularly in patients with VaD.

The association of SDMA with dementia remains largely unexplored. Little is known on SDMA in AD and nothing in VaD. Previously, Arlt *et al*.^11^ reported significant SDMA elevation in CSF from AD patients, accompanied by its insignificant increase in plasma. Lack of significant changes in AD as compared to controls has been reported also by others^15,16^. Here, SDMA was increased in dementia but the difference lost its significance following age-adjustment, implying the association of SDMA with age rather than dementia. Similarly to ADMA, SDMA accumulation was associated with unfavorable characteristics as it directly correlated with the degree of cognitive impairment and was particularly strongly associated with mental decline in patients with strategic infarcts. Corroborating our findings, SDMA was associated both with subjective and objective memory loss in older individuals in a population-based study^22^. Here, SDMA positively correlated with the degree of brain atrophy but the association seemed to be mediated by DMA, with which SDMA was positively correlated. The SDMA was also positively, although weakly, correlated with brain ischemia. These results correspond well with previous observation on SDMA being an independent predictor of adverse clinical outcomes^31^ and all-cause mortality^30^ in patients with acute stroke, irrespectively of renal function.

The DMA status in dementia was unknown. To the best of our knowledge, only Mulder *et al*.^16^, assessed DMA together with arginine, ADMA and SDMA in CSF from AD patients. Although not significantly, DMA has been the only metabolite found to be increased. Here, DMA was increased in sera from demented patients but, similarly to SDMA, the difference lost significance following the adjustment to potential confounders. However, DMA accumulation was an independent predictor of the degree of global cortical atrophy. The DMA was also increased in patients with strategic infarcts in whom it reflected medial temporal lobe atrophy and particularly strongly correlated with brain ischemia. Little is known on clinical relevance of DMA but mechanistically, DMA exposure has been associated with inflammatory responses and metaplasia in challenged epithelium^37^, neurotoxic effects^37^, and the synthesis of carcinogenic nitrosamines^38^. In line with previous findings showing that DMA positively correlates with ADMA in urine^39^, what is believed to reflect DMA synthesis from ADMA via DDAH catalysis, also serum concentrations of these metabolites positively correlated in both healthy individuals and patients with dementia. However, SDMA and not ADMA was an independent predictor of DMA concentrations, stronger among VaD than AD patients, what may reflect pro-inflammatory character of both SDMA and DMA. SDMA has been shown to correlate with IL-6 and MCP-1 after acute stroke while the rise in ADMA concentrations corresponded with markers of extracellular matrix degradation^40^. Also, ADMA is not the only source of DMA as the metabolite can be obtained from diet as well as through the activity of gut microbiota^38^.

Citrulline, similarly to arginine, was decreased in dementia and was its independent predictor. Moreover, it was decreased in all types of dementia as compared to healthy individuals but was substantially lower in patients with strategic infarcts. In arginine/NO pathway, citrulline is synthesized by both NOS and DDAH enzymes (together with DMA). Inflammation and oxidative stress hamper eNOS and DDAH activity. However, the lack of accumulation of ADMA and rather increased concentrations of DMA do not support DDAH down-regulation as a cause of diminished citrulline. On the contrary, we observed that in patients with strategic infarcts, the level of citrulline, like DMA, was significantly elevated, implying that the activity of DDAHs in those patients might be up-regulated. Correspondingly, citrulline accumulation in CSF in association with multi-infarct dementia, but not neurodegenerative one, has been reported^41^. Concomitant decrease in arginine concentrations suggests that diminished citrulline might result from down-regulated eNOS and/or enhanced citrulline utilization for NO synthesis. Correspondingly, the interventions with citrulline have been reported to be more effective in increasing NO than those with arginine^42^. Of all measured metabolites, only ADMA was independently associated with citrulline. However, the concentrations of both metabolites were interrelated only in AD.

Concentrations of metabolites of arginine/NO pathway might be affected by nutritional factors. While obesity is an unquestioned risk factor for VaD, as confirmed here by BMI being an independent predictor of VaD, the association between BMI and AD remains controversial^43^. Nonetheless, dementia is frequently accompanied by a change of nutritional habits and weight loss as well as nutritional deficits are common in dementia and parallel the severity of cognitive impairment^44^. Our patients, however, were well nourished as indicated by BMI, slightly elevated, and relatively high MNA score, similar in all patients. An association between BMI and the metabolites of NO pathway was observed only for citrulline, ADMA, and SDMA and exclusively in patients with mixed dementia and solely arginine tended to reflect decreasing MNA. Inverse relationship between citrulline and BMI corresponds well with earlier observation on diminished metabolite in obese individuals^45^. Arginine depletion might be attributed to the up-regulation of iNOS, and thus increased utilization of arginine, demonstrated in both AD and VaD^46^. Moreover, arginases, enzymes of the alternative pathway for arginine utilization, may also contribute. Their up-regulated expression and the increased concentrations of polyamines, synthesized from arginase-derived ornithine, have been observed in animal models of AD^47^. Noteworthy, the overexpression of arginases and relative arginine depletion uncouple the eNOS, causing the enzyme to switch to the production of superoxide anion, thus leading to the oxidative stress in the endothelium^48^.

As both novel biomarkers and treatment modalities are desperately needed^2^, we evaluated the strength of observed associations to appraise the potential of intermediates in NO metabolism as possible biomarkers in dementia. As we have previously demonstrated^49–51^, multiplexing several markers might improve the diagnostic power of an assay as compared to the individual measurements of its components. Here, simultaneous determination of arginine and citrulline substantially improved both their overall accuracy and specificity as dementia markers as compared to individual assessments. Moreover, arginine and citrulline significantly contributed to the model discriminating demented patients from controls with superior 94% accuracy.

The important limitation of our study is a disparity in age between groups, which we addressed by verifying all observations in the analyses including age and other potential confounders. Due to natural history of neurodegenerative diseases, finding an age-matched cohort of healthy individuals, not only without mental deficits but also without cardiometabolic diseases, known to affect the NO/arginine pathway^52^, is a challenge. Here, we enrolled blood donors, which, however, were younger due to age restrictions for blood donation. Therefore, a group of 12 patients was additionally included, in whom dementia, mild cognitive impairment or severe somatic diseases were excluded, but who were diagnosed, including neuroimaging, along our demented patients due to reported complaints (problems with memory, unexplained dizziness, headaches, etc.). As demonstrated here, those patients had already altered concentrations of metabolites of NO/arginine pathway as compared to healthy blood donors, that is, lower arginine, citrulline, and ADMA, which could not be attributed to the age difference between groups.

## Conclusions

Metabolites in arginine/NO pathway are differently altered in dementia - while arginine, Arg/ADMA, ADMA and citrulline are decreased, SDMA and DMA are elevated but their increase is associated with old age rather than dementia. Nonetheless, in patients with dementia, alterations in all these metabolites reflect unfavorable characteristics: degree of brain tissue loss and ischemia and severity of cognitive impairment. Pathology of dementia affects metabolite concentrations and their association pattern with neurodegenerative and vascular markers of brain damage and the degree of cognitive loss.

## Materials and Methods

### Study population

Study population comprised 262 individuals: 122 with dementia and 140 without dementia. Patients with dementia were recruited from the Alzheimer Center, Wroclaw Medical University, Ścinawa, Poland between December 2009 and April 2011. Inclusion criteria were age over 45 years, otherwise unremarkable medical history, no ongoing infection, and willingness to participate. Exclusion criteria were brain tumors or other malignancies detected during the diagnostic process, other neurodegenerative disorders, mild cognitive impairment, hypothyroidism, alcoholism, and unfinished diagnostic process. All patients were submitted to the evaluation of their medical history (questions about education, cognitive deficits (time of their appearance, course, progression), changes in professional, social and family functioning, habits, addictions, mood changes, presence of psychotic symptoms, other diseases (past or present) and medications) and to the internal, neurological, psychiatric, and neuropsychological examination as well as neuroimaging (computed tomography (CT) and magnetic resonance imaging (MRI)). Electrocardiogram, electroencephalogram and routine blood work including blood morphology with smear, erythrocyte sedimentation rate, electrolytes, urea, creatinine, asparagine and alanine transaminases, thyroid-stimulating hormone (TSH), urinalysis, lipid/lipoprotein profile, and in certain cases, tests for HIV, syphilis, Lyme disease, or other diseases were conducted as well. Among the enrolled patients, 48 were diagnosed with AD, 34 with MD, and 40 with VaD, according to the following criteria: Diagnostic and Statistical Manual of Mental Disorders (DSM)-IV^53^ and National Institute of Neurological and Communicative Disorders and Stroke and the Alzheimer’s Disease and Related Disorders Association (NINCDS-ADRDA)^54^ for AD, ICD-10^55^ with the Hachinski Ischemic Scale (HIS)^56^ for MD, and International Statistical Classification of Diseases and Related Health Problems (ICD-10)^55^ and National Institute of Neurological Disorders and Stroke and Association Internationale pour la Recherché et l’Enseignement en Neurosciences (NINDS-AIREN)^57^ for VaD. The MRI was used to establish the pathology of dementia and to assess the degree of structural brain changes. The 1 and 1.5 MRI scanners were used to perform imaging without contrast (in justified cases with contrast) in T1 and T2-weighted images, in SE, FSE, FLAIR, DWI sequences, in sagittal, frontal and transverse planes. The following scales were applied: the Global Cortical Atrophy (GCA) scale, the Medial Temporal Lobe Atrophy (MTA) scale, and the Fazekas scale for white matter lesions^58^. Computed tomography was applied in patients suffering from claustrophobia and/or with pacemakers or other metal elements in their bodies. The MRI and CT scans were evaluated by an independent, experienced and blinded radiologist. Deterioration of cognitive function was determined using the Mini Mental State Examination (MMSEs) scales^59^ as well as the Clinical Dementia Rating (0–5 scale)^60^. Patients’ nutritional status was evaluated using the Mini Nutritional Assessment (MNA; 14-point scale)^61^ and the body mass index (BMI).

Control group consisted of 128 blood donors who were recruited from the Regional Center for Blood Donation and Blood Therapeutics in Wroclaw, Poland. The following inclusion criteria were applied: age >45 yrs, unremarkable general medical exam, normal blood count without any evidence of anemia or inflammation, and no complaints on memory and cognitive function. Additionally, 12 patients who underwent MRI examination due to memory loss complain or unexplained dizziness or headaches but without dementia, mild cognitive impairment, or significant somatic diseases were included.

Dementia and control group of blood donors did not differ with respect to sex distribution (Table 1) but there was significant difference in age due to natural history of dementia and the fact that individuals 65+ are not suitable for blood donation. Therefore, the age difference was accounted for in the statistical analysis.

### Ethical considerations

The study protocol was approved by the Medical Ethics Committee of Wroclaw Medical University (KB-679/2011 and KB-367/2017) and the study was conducted in accordance with the Helsinki Declaration of 1975, as revised in 1983, and informed consent was obtained from all study participants. In the case of patients with severe dementia, an informed consent for study participation has been obtained from legal guardians.

### Analytical methods

#### Samples from patients

Blood samples were drawn following overnight fasting into serum-separator tubes by venipuncture, clotted for 30 min, and subsequently centrifuged (10 min, 1500 × g). Resulting sera were collected, aliquoted and stored frozen at −80 °C.

#### Chemicals

Benzoyl chloride (BCl), hydrochloride salts of unlabeled dimethylamine (DMA), hexadeutero-dimethylamine (D6-DMA, 99%), L-arginine, SDMA, ADMA, L-citrulline, and sodium tetraborate were procured from Sigma-Aldrich (Poznan, Poland). Isotope labeled L-arginine:HCl (D7-arginine, 98%) and asymmetric dimethylarginine (2,3,3,4,4,5,5-D7-ADMA, 98%) were obtained from Cambridge Isotope Laboratories (Tewksbury, MA, USA). Methanol, acetonitrile, water, and formic acid were acquired from Merck Millipore (Warsaw, Poland), and leucine–enkephalin was from Waters (Milford, MA, USA).

#### Sample preparation

Patients’ serum samples and calibration standards were prepared in the same manner according to the previously published method^8^. Briefly, 100 µL aliquots of calibration standards or serum, 10 µL of internal standard solution (50 µM D6-DMA, 20 µM D7-ADMA, and 100 µM D7-arginine, respectively) and 50 µL of borate buffer (0.025 M Na_2_B_4_O_7_ × 10H_2_O, 1.77 mM NaOH, pH = 9.2) were placed into 2.0 mL polypropylene tubes and vortexed (1 min, 25 °C). Derivatization was conducted using 400 µL of acetonitrile (ACN) and 10 µL of 10% BCl in ACN. The solutions were incubated and vortexed (5 min, 25 °C), centrifuged (7 min, 10,000 RPM, 4 °C), and 100 µL of the clear supernatant was transferred into glass vials containing 400 µL of water.

Standard calibration curves were prepared using the following concentration ranges: 5 to 250 µM for arginine, 0.05 to 2.5 µM for ADMA and SDMA, 1 to 50 µM for citrulline, and 0.14 to 7.0 µM for DMA.

#### Analytical chromatography

Analytical chromatography was conducted using Acquity UPLC system equipped with cooled autosampler (Waters, Milford, MA, USA) and Acquity HSS T3 column (50 × 1.0 mm, 1.75 µm) from Waters. Elution was carried out with 0.1% formic acid (FA) in water and 0.1% FA in methanol as a mobile phase A and B, respectively. Total run time was 10 min with total flow rate of 250 µL/min. The following gradient was applied: 5% B for 0–0.5 min, 5%–14% B for 0.5–3 min, 14%–60% B for 3–4 min, 60%–90% B for 4–4.5 min, 90% B for 4.5–5 min and 90%–5% B for 5–5.10 min.

#### Mass spectrometry

Mass spectrometric analysis was conducted using Xevo G2 Quadrupole TOF MS (Waters, Milford, MA, USA) with electrospray ionization (ESI) in positive ion mode. The spray voltage, source temperature and the desolvation temperature were set at 0.5 kV, 120 °C and 450 °C, respectively. Nitrogen was used as the nebulizing and drying gas. Data were acquired by using MassLynx software (Waters) for the following ions (m/z): 279.1457, 286.1897, 307.1770, 314.2209, 280.1297, 150.0919, and 156.1295 for arginine, D7-arginine, ADMA, SDMA, D7-ADMA, citrulline, DMA, and D6-DMA, respectively.

### Statistical analysis

Data distribution was analyzed using Kolmogorov-Smirnov test and homogeneity of variances using Levene’s test. Normally distributed data (after log 10-transformation if necessary) are presented as means (geometric mans) with 95% confidence interval (CI) and categorical data as medians with interquartile range (IQR). Depending on data character, 1-way ANOVA with post-hoc Tukey-Kramer test or Kruskal-Wallis H test with Conover post-hoc test (for multigroup comparisons) and t-test for independent samples or t-test with Welch correction in case of unequal variances (for two-group comparisons) were applied. Frequency analysis was conducted using χ^2^ test and correlation analysis using the Pearson (r) or Spearman (ρ) tests. Confounder-adjusted analysis was conducted using analysis of co-variance (ANCOVA). Multiple linear regression (stepwise method; variables were entered into the model if p < 0.05 and removed if p > 0.1) was used to discern independent predictors of explained (dependent) continuous variable. Correlation coefficients (b) and partial correlation coefficients (r_p_) were calculated for independent variables and the goodness-of-fit of the model was expressed as % of explained variance (coefficient of determination R^2^ in percent). Logistic regression (stepwise method; variables were entered into the model if p < 0.05 and removed if p > 0.1) was used to discern independent predictors of explained (dependent) dichotomous variable. Correlation coefficients (b) were calculated for independent variables and the goodness-of-fit of the model was expressed as % of correctly classified cases. Additionally, calculated probabilities of selected models were used for constructing the receiver operating characteristics (ROC) curves. The overall accuracy of a model was expressed as an area under the ROC curve (AUC). The optimal cut-off values were determined as well and given with corresponding sensitivities and specificities. A two-tailed probability of < 0.05 was considered significant. The analyses were performed using MedCalc Statistical Software version 19.0.5 (MedCalc Software bvba, Ostend, Belgium; https://www.medcalc.org; 2019).

## Supplementary information

Supplementary data 1

## Data Availability

The datasets generated during and/or analysed during the current study are available from the corresponding author on reasonable request.
