# Supplementary material for: Targeted metabolomic analysis of nitric oxide/L-arginine pathway metabolites in dementia: association with pathology, severity, and structural brain changes
Source: Sci Rep. 2019 Sep 24;9:13764. doi: 10.1038/s41598-019-50205-0 (PMC6760237; doi:10.1038/s41598-019-50205-0)
Supplement: Supplementary file 1 — Supplementary data 1 [file 41598_2019_50205_MOESM1_ESM.pdf]

Title: Targeted metabolomic analysis of nitric oxide/L-arginine pathway metabolites in dementia: association with pathology, severity, and structural brain changes

Authors: Mariusz G. Fleszar, Jerzy Wiśniewski, Marzena Zboch, Dorota Diakowska, Andrzej Gamian, Małgorzata Krzystek-Korpacka

Supplementary Figure S1 The correlation between DMA and the degree of medial temporal lobe atrophy (MTA) in a subgroup of dementia patients with strategic infarcts

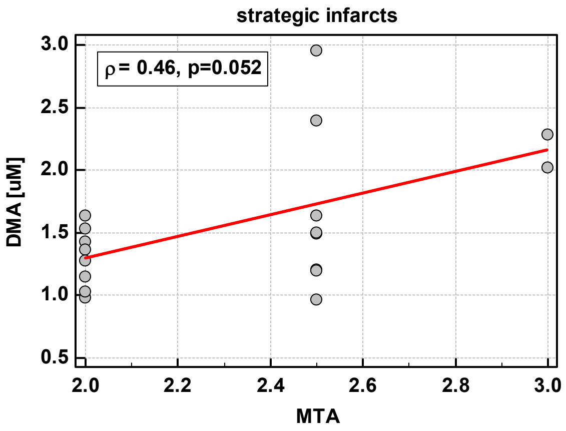

Data analyzed using Spearman rank test and presented as correlation coefficient rho (ρ).

Supplementary Figure S2. Correlograms showing the interrelationship between metabolites of arginine/No pathway depending on analyzed group

a. Controls: blood donors

1.0 -1.0

	log(sdma)	log(adma)	log(dma)	log(cit)	log(Arg)
log(sdma)		0.514	0.589	0.176	-0.230
log(adma)	0.514		0.378	0.241	-0.070
log(dma)	0.589	0.378		0.095	-0.231
log(cit)	0.176	0.241	0.095		-0.190
log(Arg)	-0.230	-0.070	-0.231	-0.190	

b. Controls: non-demented patients

1.0 -1.0

	log(sdma)	log(adma)	log(dma)	log(cit)	log(Arg)
log(sdma)		0.922	0.870	0.708	0.612
log(adma)	0.922		0.845	0.704	0.566
log(dma)	0.870	0.845		0.695	0.645
log(cit)	0.708	0.704	0.695		0.457
log(Arg)	0.612	0.566	0.645	0.457	

c. Dementia patients: all

1.0 -1.0

	log(sdma)	log(adma)	log(dma)	log(cit)	log(Arg)
log(sdma)		0.512	0.551	0.256	0.025
log(adma)	0.512		0.190	0.297	0.249
log(dma)	0.551	0.190		0.083	-0.019
log(cit)	0.256	0.297	0.083		0.189
log(Arg)	0.025	0.249	-0.019	0.189	

d. Dementia patients: Alzheimer disease

1.0 -1.0

	log(adma)	log(sdma)	log(cit)	log(dma)	log(Arg)
log(adma)		0.518	0.500	0.102	0.283
log(sdma)	0.518		0.197	0.399	0.142
log(cit)	0.500	0.197		-0.054	-0.015
log(dma)	0.102	0.399	-0.054		0.051
log(Arg)	0.283	0.142	-0.015	0.051	

e. Dementia patients: mixed-type dementia

1.0 -1.0

	log(sdma)	log(dma)	log(adma)	log(cit)	log(Arg)
log(sdma)		0.785	0.662	0.412	0.037
log(dma)	0.785		0.572	0.225	-0.139
log(adma)	0.662	0.572		0.212	0.246
log(cit)	0.412	0.225	0.212		0.454
log(Arg)	0.037	-0.139	0.246	0.454	

f. Dementia patients: vascular dementia

1.0 -1.0

	log(sdma)	log(dma)	log(adma)	log(cit)	log(Arg)
log(sdma)		0.733	0.489	0.231	-0.014
log(dma)	0.733		0.244	0.239	0.085
log(adma)	0.489	0.244		0.204	0.150
log(cit)	0.231	0.239	0.204		0.189
log(Arg)	-0.014	0.085	0.150	0.189	

Data presented as Pearson correlation coefficients.

Supplementary Table S1. Variables independently associated with intermediates in NO metabolism (multiple regression)

Dependent variable	Healthy controls	Dementia-AD	Dementia-VaD
**Arginine (Arg)**	**DMA**: r_P=-0.23, p=0.012<br>R^2=0.05	no variables retained	no variables retained
**ADMA**	**SDMA**: r_P=0.53, p<0.0001<br>R^2=0.28	**Arg**: r_P=0.31, p=0.039<br>**SDMA**: r_P=0.48, p<0.001<br>**Cit**: r_P=0.50, p<0.001<br>R^2=0.49	**SDMA**: r_P=0.49, p=0.001<br>R^2=0.24
**SDMA**	**ADMA**: r_P=0.41, p<0.0001<br>**DMA**: r_P=0.49, p<0.0001<br>R^2=0.45	**ADMA**: r_P=0.52, p<0.001<br>**DMA**: r_P=0.41, p=0.005<br>R^2=0.39	**ADMA**: r_P=0.47, p=0.003<br>**DMA**: r_P=0.73, p<0.0001<br>R^2=0.64
**Citrulline (Cit)**	**ADMA**: r_P=0.25, p=0.006<br>R^2=0.06	**ADMA**: r_P=0.50, p<0.001<br>R^2=0.25	no variables retained
**DMA**	**SDMA**: r_P=0.59, p<0.0001<br>R^2=0.34	**SDMA**: r_P=0.40, p=0.005<br>R^2=0.15	**SDMA**: r_P=0.73, p<0.0001<br>R^2=0.54

Results of multiple regression (stepwise method) presented as variables retained in the model (in bold) together with partial correlation coefficient (r_P; net correlation, without the interference of others) and statistical significance as well as goodness of fit of the model represented by the coefficient of determination R^2.
